# Randomized Controlled Trial of the Immunogenicity and Safety of a Serum-Free Purified Vero Rabies Vaccine (PVRV-NG2) Using a Simulated Postexposure Zagreb Regimen With Human Rabies Immunoglobulin in Adults in Thailand

**DOI:** 10.1093/ofid/ofae633

**Published:** 2024-10-25

**Authors:** Danaya Chansinghakul, Terapong Tantawichien, Kriengsak Limkittikul, Winai Ratanasuwan, Yuancheng Wang, Celine Petit, Francoise Guinet-Morlot, Carina Frago, Andrea-Clemencia Pineda-Peña

**Affiliations:** Global Clinical Development Strategy, Sanofi, Bangkok, Thailand; Faculty of Medicine, Chulalongkorn University, Bangkok, Thailand; Queen Saovabha Memorial Institute, The Thai Red Cross Society, Bangkok, Thailand; Faculty of Tropical Medicine, Mahidol University, Bangkok, Thailand; Faculty of Medicine, Siriraj Hospital, Mahidol University, Bangkok, Thailand; Global Biostatistical Sciences, Sanofi, Chengdu, China; Global Immunology, Sanofi, Marcy l’Étoile, France; New Vaccine Portfolio/Project Strategy & Execution, Sanofi, Marcy l’Étoile, France; Global Clinical Development Strategy, Sanofi, Singapore; Global Clinical Development Strategy, Sanofi, Marcy l’Étoile, France

**Keywords:** immunogenicity, postexposure prophylaxis, rabies, safety, vaccine

## Abstract

**Background:**

A serum-free, highly purified Vero rabies vaccine–next generation (PVRV-NG2) is under development. We conducted a phase III trial to describe the safety and immunogenicity profile of PVRV-NG2 compared with those of licensed purified Vero rabies vaccine (PVRV) in a simulated rabies postexposure prophylaxis (PEP) Zagreb regimen in Thailand.

**Methods:**

Healthy adults aged ≥18 years (n = 201) were randomized in a 2:1 ratio to receive PVRV-NG2 or PVRV in a rabies PEP Zagreb (days 0, 7, 21 [2-1-1]) regimen, with concomitant human rabies immunoglobulin (HRIG) at day 0. Immunogenicity end points included the proportion of participants with rabies virus–neutralizing antibody (RVNA) titers ≥0.5 IU/mL at days 0, 14, and 35. Safety outcomes were also assessed.

**Results:**

A total of 199 participants completed the study (PVRV-NG2 n = 133, PVRV n = 66). In the PVRV-NG2 group and PVRV group, respectively, 91.0% (95% CI, 84.1%–95.6%) and 94.6% (95% CI, 85.1%–98.9%) had RVNA titers ≥0.5 IU/mL at day 14, increasing to 100% (95% CI, 96.8%–100%) and 100% (95% CI, 93.5%–100%) by day 35. The vaccines had similar safety profiles, and there were no safety concerns.

**Conclusions:**

PVRV-NG2 showed acceptable safety and immunogenicity profiles when co-administered with HRIG in a simulated PEP Zagreb regimen in healthy adults in Thailand.

## Graphical Abstract

**Graphical Abstract:**
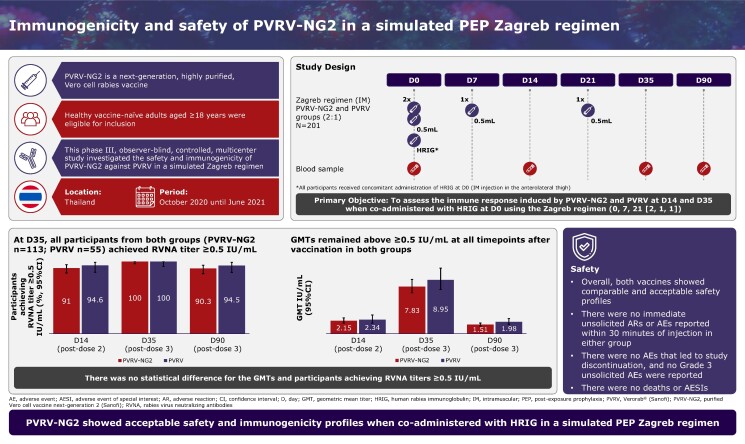

## Teaser

A serum-free, highly-purified Vero rabies vaccine-next generation, PVRV-NG2, showed acceptable safety and immunogenicity profiles, comparable to those of licensed purified Vero rabies vaccine, in a simulated post-exposure prophylaxis Zagreb regimen with concomitant human rabies immunoglobulin administration on Day 0.

According to the World Health Organization (WHO), rabies causes 59 000 human deaths annually across 150 countries, most of which occur in Africa and Asia; 99% of human rabies cases are dog-mediated [1]. Rural populations are disproportionately affected, and ∼40% of dog-mediated cases of rabies are among children aged 4–15 years [1, 2].

Rabies is endemic in 8 of 10 Southeast Asian countries, with 600 million people at risk in Cambodia, Indonesia, Laos, Malaysia, Myanmar, the Philippines, Thailand, and Vietnam [3]. In Thailand from 2010 to 2015, there were 46 confirmed or probable cases of rabies [3]. Eleven of these cases were reported in Eastern Thailand, where there were 6204 suspected rabies exposures during 2015, most of which involved dogs (77.8%) [3, 4].

The main strategy for protection against rabies in humans is vaccination, either as a pre-exposure prophylaxis (PrEP) regimen in those at high risk of exposure or as a postexposure (PEP) regimen [2]. Although the WHO does not currently recommend rabies vaccination for PrEP in its Expanded Program on Immunization [2], the Pediatric Infectious Disease Society of Thailand recommends a PrEP rabies vaccine using 2 doses with at least a 7-day interval starting from the age of 2 months for at-risk populations [5].

PEP consists of rabies vaccination and a dose of human rabies immunoglobulin (HRIG) on the day of exposure and further vaccine doses up to 3 weeks postexposure [2]. The Zagreb regimen includes 4 doses (0.5 mL each) of rabies vaccine, 2 of which are given on the day of exposure (1 in each deltoid), with the third and fourth doses administered 7 and 21 days postexposure, respectively [6]. The Essen regimen includes either 4 doses (0.5 mL each) of rabies vaccine, with 1 dose on the day of exposure and subsequent doses 3, 7, and 14–28 days postexposure (recommended by the WHO), or 5 doses (0.5 mL each), with 1 administered on the day of exposure and the others 3, 7, 14, and 28 days postexposure [2, 7]. The Essen regimen is widely used worldwide, but some countries still use the Zagreb regimen [8]. Several vaccines have been used successfully in PrEP and PEP regimens for decades; among these, the human diploid cell vaccine (HDCV; Imovax Rabies, Sanofi) and purified Vero cell rabies vaccine (PVRV; Verorab, Sanofi) have well-defined safety and immunogenicity profiles [9, 10].

A next-generation, serum- and antibiotic-free, highly purified, freeze-dried Vero cell rabies vaccine candidate (PVRV-NG) was developed using the same Pitman-Moore viral strain as the already licensed HDCV and PVRV rabies vaccines. The immunogenicity and safety profiles of PVRV-NG have been described in PrEP and PEP regimens in several phase II and III clinical trials [11–15]. The PVRV-NG vaccine was later reformulated to PVRV-NG2 following a phase II dose-ranging study conducted in the United States [14]. This study evaluated a simulated PEP 5-dose Essen regimen with HRIG and showed that the highest PVRV-NG2 dose (7.6 IU/dose) elicited immune responses consistent with those of the licensed HDCV rabies vaccine, with higher geometric mean titers (GMTs) and geometric mean titer ratios (GMTRs) at each time point, a similar proportion of participants displaying rabies virus–neutralizing antibody (RVNA) titers ≥0.5 IU/mL, and fewer reports of adverse reactions [14]. Furthermore, a phase III study reported the immunological noninferiority at day (D) 28 of PVRV-NG2 compared with licensed PVRV and HDCV using the simulated PEP 5-dose Essen regimen with co-administration of HRIG at D0 in healthy adults in France [16].

In this phase III, observer-blind study, we describe the immunogenicity and safety of PVRV-NG2 vaccine compared with those of a licensed PVRV vaccine administered as a PEP Zagreb regimen in adults in Thailand.

## METHODS

This phase III, observer-blind, multicenter study was conducted at 3 centers in Thailand from October 11, 2020, to June 23, 2021. Healthy adults received PVRV-NG2 or PVRV (Verorab, Sanofi) according to a PEP Zagreb schedule with a 6-month follow-up period (ClinicalTrials.gov Identifier: NCT04594551; WHO: U1111-1238-1726).

Participants were eligible for inclusion if they were healthy, aged ≥18 years, and had not received any previous PrEP or PEP vaccination against rabies. Key exclusion criteria were any vaccination in the previous 4 weeks; receipt of any immunoglobulins, blood, or blood-derived products within the previous 3 months; and being either bitten by or exposed to a potentially rabid animal within the previous 6 months or at high risk for rabies exposure.

### Patient Consent

The study was conducted in accordance with the protocol and consensus ethical principles derived from international guidelines including the Declaration of Helsinki, the International Council for Harmonisation of Technical Requirements for Pharmaceuticals for Human Use Guidelines for Good Clinical Practice, and all applicable laws, rules, and regulations. The study was reviewed and approved by an independent ethics committee/institutional review board at each site. The authorizing bodies were the Ethics Committee of the Faculty of Tropical Medicine, Mahidol University, Bangkok, Thailand; the Institutional Review Board of the Faculty of Medicine, Chulalongkorn University, Bangkok, Thailand; and the Siriraj Institutional Review Board of the Faculty of Medicine Siriraj Hospital, Bangkok, Thailand. All participants provided informed consent by means of a written, signed, and dated informed consent form before any study procedures were performed.

### Vaccines and HRIG

PVRV-NG2 and PVRV (Verorab, Sanofi, France) were derived from the same Wistar rabies virus Pitman Moore/WI 38 1503-3 M strain. Tested batches of each vaccine fulfilled the National Institutes of Health potency of ≥2.5 IU per dose [17]. The actual antigen content of each vaccine was also measured by enzyme-linked immunosorbent assay, as previously described [18, 19]. The antigen content was 8.0 IU/dose (batch number: S4497) for PVRV-NG2 and 3.1 IU/dose (batch number: R1F31) for PVRV. Vaccines were provided as freeze-dried powders and were reconstituted immediately before use with 0.5-mL saline diluent. Both vaccines were produced according to Good Manufacturing Practices standards.

Rabies-HT HRIG in liquid/solution (IMOGAM, Sanofi, France) was used at the recommended PEP dosage of 20 IU/kg body weight (calculation based on the 150-IU/mL specification, but the potency by Rapid Focus Fluorescent Inhibition Test [RFFIT] was 202 IU/mL).

### Schedule and Procedures

Eligible participants were randomized using Interactive Response Technology, with permuted block randomization and stratification by center, in a 2:1 ratio to the PVRV-NG2 and PVRV groups. Unblinded staff members, independent of the safety evaluation and other study evaluations, prepared and administered the vaccine. Participants, investigators, staff members in charge of safety assessments, and sponsor staff were blinded to participant group.

**Figure 1.**
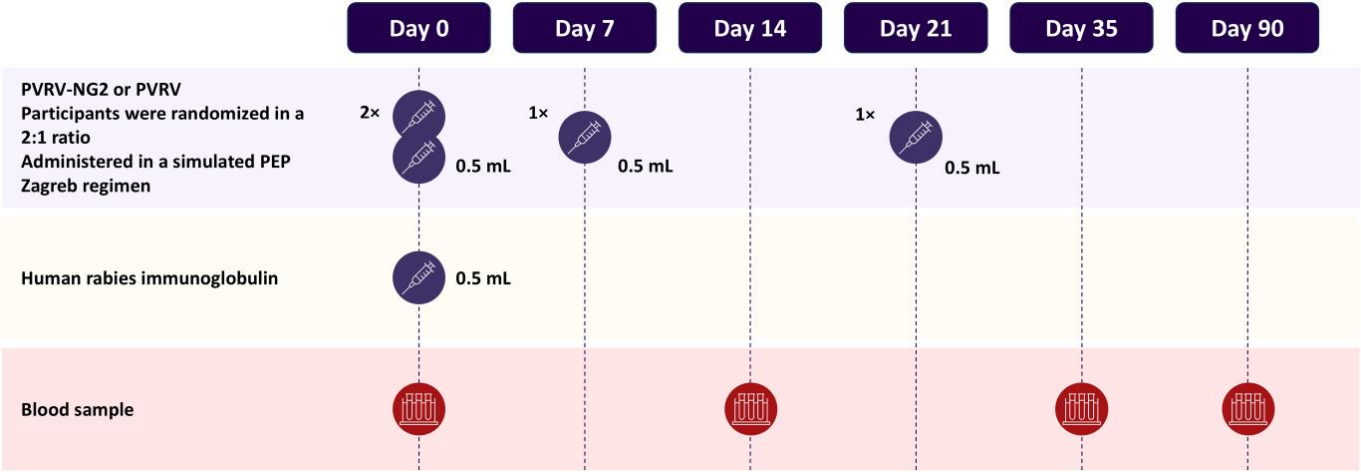
Study design until day 90. A safety follow-up phone call was performed at 6 months (not shown here). Abbreviations: PEP, postexposure prophylaxis; PVRV, purified Vero cell rabies vaccine; PVRV-NG2, next-generation purified Vero cell rabies vaccine.

The study schedule is shown in Figure 1. A total of 7 visits were planned during the first 90 days, with a safety follow-up phone call at 6 months. Baseline demographic data were collected from all participants on D0 (visit 1). Participants received 4 intramuscular (IM) injections in line with the Zagreb regimen: The first 2 doses (0.5 mL) were given by 2 IM injections in each deltoid on D0 (visit 1), the second dose was administered as a 0.5-mL injection at D7 (visit 2), and the third dose as a 0.5-mL injection at D21 (visit 4). All participants received concomitant administration of HRIG at D0. HRIG was administered IM in the anterolateral thigh. A maximum of 5.0 mL of HRIG was injected, with the total volume divided and administered at separate sites (at least 3 cm apart).

For immunogenicity assessments, blood samples were collected during study visits, before vaccination and HRIG on D0, and on D14 (7 days after second vaccination), D35 (14 days after third vaccination), and D90 (3 months after first vaccination). Samples were shipped frozen, using dry ice to maintain them in a frozen state, in the packaging container provided by the carrier. Samples were assessed at the Sanofi Pasteur serology laboratory (Global Clinical Immunology [GCI], Swiftwater, PA, USA).

### Objectives

The primary objective was to describe the immune responses at D14 and D35 elicited by PVRV-NG2 and PVRV administered using a simulated PEP Zagreb regimen with HRIG on D0. Secondary objectives were to describe the immune responses at D90 and to describe the tolerability and safety profile of each vaccine.

### Immunogenicity

The co-primary immunogenicity end points were the proportion of participants with an RVNA titer ≥0.5 IU/mL at D0, D14, and D35; the proportion of participants with an RVNA titer ≥ the lower limit of quantification (LLOQ; 0.2 IU/mL) at D0, D14, and D35 (if a value < LLOQ was recorded, then a value of 0.1 IU/mL [LLOQ/2] was used in the calculation); and individual RVNA titer ratios: D14/D0 and D35/D0. Secondary immunogenicity end points were the proportion of participants with an RVNA titer ≥0.5 IU/mL at D90, participants with an RVNA titer ≥ LLOQ at D90, and individual RVNA titer ratios (D90/D0). Antibody titers were also described as GMTs at each time point, and the GMTR was calculated as the GMT at each time point compared with the D0 GMT.

RVNA titers were measured using the RFFIT at the Sanofi GCI laboratory [20]. Briefly, rabies virus–specific antibodies present in serum were assessed with a standardized challenge dosage of rabies virus (CVS-11) in a micro-neutralization cell culture. Non-neutralized rabies virus in the serum/virus mixture was detected in the infected cells by a direct fluorescence antibody method using fluorescein isothiocyanate–conjugated antirabies monoclonal immunoglobulin. The rabies virus in micro-neutralization cell cultures was enumerated in scanned images generated from a cell imaging reader. The absence of infectivity (no fluorescent cells) constituted a positive neutralization reaction, indicating the presence of RVNAs in the serum. The infection of cells in culture indicated the absence of RVNAs in the serum. The highest dilution of the serum that neutralizes 50% of the challenge virus was the end point antibody titer. The RVNA concentration (expressed in IU/mL) was determined by calibrating the 50% neutralization end point antibody titer of the test serum to the 50% neutralization end point titer of an internal reference serum, which was calibrated against the first WHO international standard for antirabies immunoglobulin [21]. Titers (in IU/mL) were obtained in duplicate for each tested sample, and the individual geometric mean of duplicates was calculated as needed. The LLOQ for the RFFIT assay was 0.2 IU/mL. Samples calculated to a value less than the LLOQ were reported as < LLOQ.

### Safety Assessments

Solicited adverse events (AEs) within 7 days after each vaccination were recorded on diary cards by participants. These included injection site reactions (pain, swelling, and erythema) and systemic reactions (fever, headache, malaise, and myalgia). Unsolicited AEs and adverse reactions (ARs) were recorded within 30 minutes postvaccination and for 28 days after the last vaccination. ARs and AEs were defined and coded using the Medical Dictionary for Regulatory Activities [22]. Serious AEs (SAEs) and AEs of special interest (AESIs; anaphylactic reactions, encephalitis, and convulsions) were assessed throughout the study and up to 6 months after the last vaccination. Investigators graded AEs and ARs for intensity. Grade 1 was defined as no interference with usual activities, grade 2 was defined as some interference with usual activities, and grade 3 was defined as significant interference and prevention of usual activities.

### Statistical Methods

The study was designed to be descriptive. The target sample size was 201 participants allocated in a 2:1 target ratio (PVRV-NG2 n = 134, PVRV n = 67) with an assumed drop-out rate of 10% to allow the evaluation of 120 and 60 participants in the PVRV-NG2 and PVRV groups, respectively, up to D35.

Statistical analyses were performed with SAS software (SAS Institute, Cary, NC, USA), version 9.4 or higher. Baseline demographic data were reported as frequency (No.) and proportion (%) for categorical values and as mean and SD for numerical values. Point estimates and 95% CIs were calculated for all end points using the exact binomial method (Clopper-Person method) [23]. For the calculation of GMTs and GMTRs, the RVNA titers were log-transformed and calculated with a 95% CI.

Immunogenicity analyses were performed in the per-protocol analysis set (PPAS) and the full analysis set (FAS). The FAS included all randomized subjects who received ≥1 dose of a vaccine. The PPAS included all subjects in the FAS who met all protocol specifications (Supplementary Table 1). The safety analysis set (SafAS) included all participants who received ≥1 dose of vaccine.

## RESULTS

**Figure 2.**
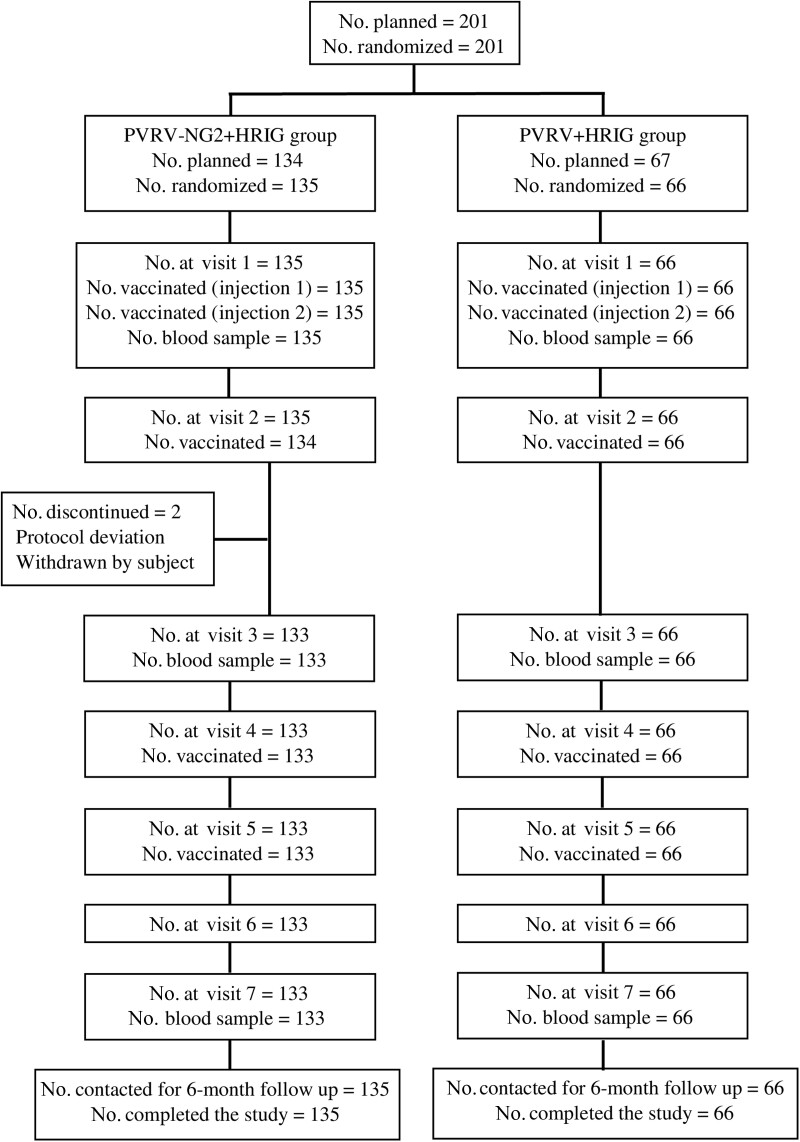
Participant disposition flowchart. Visit 6 was for the collection of safety information. Abbreviations: HRIG, human rabies immunoglobulin; No. number of participants; PVRV, purified Vero cell rabies vaccine; PVRV-NG2, next-generation purified Vero cell rabies vaccine.

A total of 201 participants were enrolled and randomized (135 to PVRV-NG2 and 66 to PVRV). All participants received 1 dose of vaccine and HRIG on D0 and were included in the SafAS (Figure 2). The vaccination schedule was completed by 133 of 135 (98.5%) participants in the PVRV-NG2 group and all 66 participants in the PVRV group. Of the 199 participants who completed the study, 167 (83.9%) and 168 (84.4%) were included in the PPAS at D14 and D35, respectively. All 201 participants were successfully contacted for the 6-month safety follow-up. Baseline demographics and characteristics were similar between the vaccine groups (Supplementary Table 2).

### Immunogenicity

**Figure 3.**
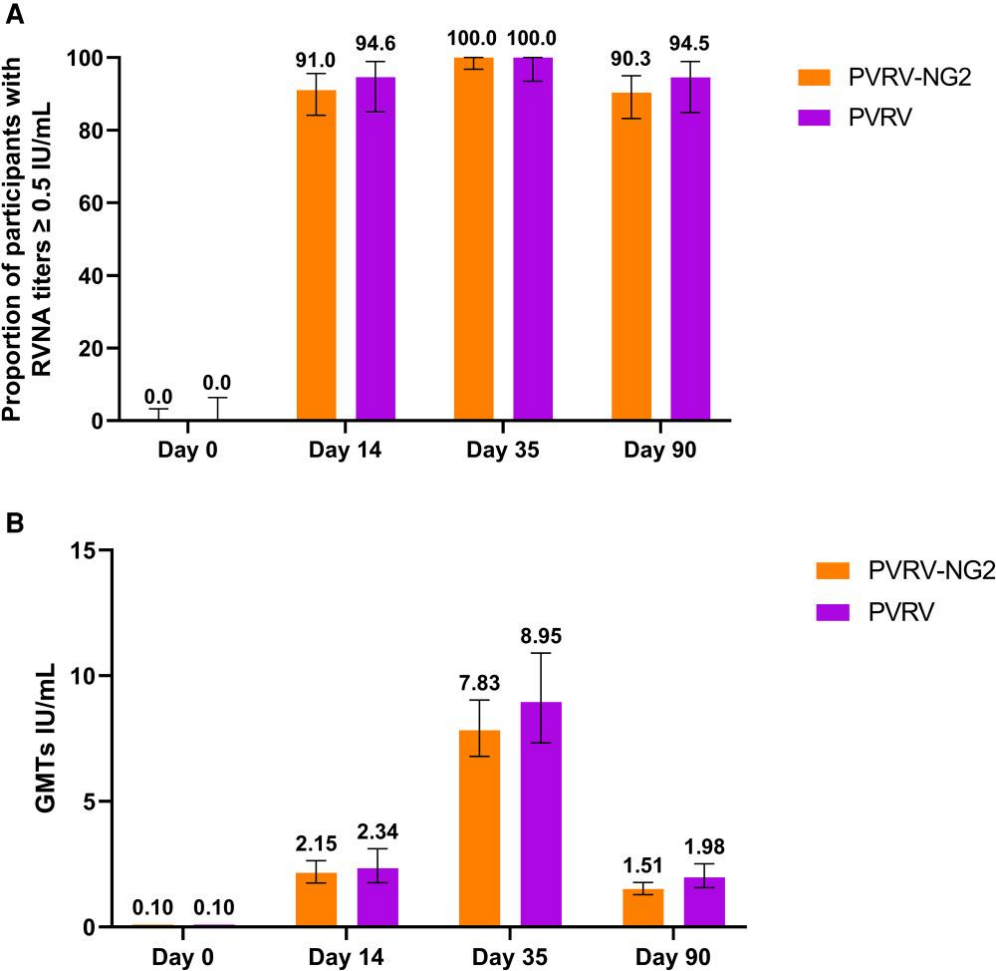
Proportion of participants achieving RVNA titers ≥0.5 IU/mL (*A*) and GMTs (*B*) in the per-protocol analysis set. Abbreviations: GMT, geometric mean titer; PVRV, purified Vero cell rabies vaccine; PVRV-NG2, next-generation purified Vero cell rabies vaccine; RVNA, rabies virus neutralizing antibody.

Overall, the immune responses in the PVRV-NG2 and PVRV groups were comparable at all time points across end points (Table 1). Before vaccination, all study participants had RVNA titers <0.2 IU/mL. The proportion of participants with RVNA titers ≥0.5 IU/mL at D14 in the PVRV-NG2 and PVRV groups was 91.0% (95% CI, 84.1%–95.6%) and 94.6% (95% CI, 85.1%–98.9%), respectively (Figure 3). At D35, the proportion of participants with RVNA titers ≥0.5 IU/mL in the PVRV-NG2 and PVRV groups was 100% (95% CI, 96.8%–100%) and 100% (95% CI, 93.5%–100%), respectively. At D90, the proportion of participants with RVNA titers ≥0.5 IU/mL in the PVRV-NG2 and PVRV groups was 90.3% (95% CI, 83.2%–95.0%) and 94.5% (95% CI, 84.9%–98.9%), respectively. The ranges of RVNA GMTs were similar between groups, with a peak after 3 doses of vaccine (Figure 3). GMTs remained ≥0.5 IU/mL at all time points after vaccination in both groups and remained ≥15-fold higher at D90 compared with D0. Similar results were observed in the FAS (Supplementary Table 3).

**Table 1.** Summary of Immunogenicity in the Per-Protocol Analysis Sets

	PVRV-NG2 (No. = 135)	PVRV (No. = 66)
D0 (before vaccination)		
Participants with available RVNA titer, No.	111	56
Participants with RVNA titer ≥0.2 IU/mL, No.	0	0
% of participants with RVNA titer ≥0.2 IU/mL (95% CI)	0 (0–3.3)	0 (0–6.4)
Participants with RVNA titer ≥0.5 IU/mL, No.	0	0
% of participants with RVNA titer ≥0.5 IU/mL (95% CI)	0 (0–3.3)	0 (0–6.4)
GMT (95% CI), IU/mL	0.100 (0.100–0.101)	0.100 (NC–NC)
RVNA titer range, IU/mL	0.100–0.141	0.100–0.100
D14 (7 d after vaccination dose 2)		
Participants with available RVNA titer, No.	111	56
Participants with RVNA titer ≥0.2 IU/mL, No.	109	55
% of participants with RVNA titer ≥0.2 IU/mL (95% CI)	98.2 (93.6–99.8)	98.2 (90.4–100)
Participants with RVNA titer ≥0.5 IU/mL, No.	101	53
% of participants with RVNA titer ≥0.5 IU/mL (95% CI)	91.0 (84.1–95.6)	94.6 (85.1–98.9)
GMT (95% CI), IU/mL	2.15 (1.75–2.64)	2.34 (1.76–3.11)
RVNA titer range, IU/mL	0.141–28.9	0.100–113.0
GMTR 7 d post–dose 2 (D14)/pre–dose 1 (D0) (95% CI)	21.4 (17.5–26.3)	23.4 (17.6–31.1)
D35 (14 d after vaccination dose 3)		
Participants with available RVNA titer, No.	113	55
Participants with RVNA titer ≥0.2 IU/mL, No.	113	55
% of participants with RVNA titer ≥0.2 IU/mL (95% CI)	100 (96.8–100)	100 (93.5–100)
Participants with RVNA titer ≥0.5 IU/mL, No.	113	55
% of participants with RVNA titer ≥0.5 IU/mL (95% CI)	100 (96.8–100)	100 (93.5–100)
GMT (95% CI), IU/mL	7.83 (6.79–9.03)	8.95 (7.33–10.9)
RVNA titer range, IU/mL	0.794–69.6	2.09–81.5
GMTR 14 d post–dose 3 (D35)/pre–dose 1 (D0) (95% CI)	78.1 (67.8–90.0)	89.5 (73.3–109)
D90 (up to 3 mo after vaccination dose 1)		
Participants with RVNA titer, No.^a^	113	55
Participants with RVNA titer ≥0.2 IU/mL, No.	111	55
% of participants with RVNA titer ≥0.2 IU/mL (95% CI)	98.2 (93.8–99.8)	100 (93.5–100)
Participants with RVNA titer ≥0.5 IU/mL, No.	102	52
% of participants with RVNA titer ≥0.5 IU/mL (95% CI)	90.3 (83.2–95.0)	94.5 (84.9–98.9)
GMT (95% CI), IU/mL	1.51 (1.29–1.77)	1.98 (1.56–2.52)
RVNA titer range, IU/mL	0.100–13.3	0.300–53.5
GMTR up to 3 mo post–dose 3 (D90)/pre–dose 1 (D0) (95% CI)	15.1 (12.9–17.6)	19.8 (15.6–25.2)

Abbreviations: d, days; D, day; GMT, geometric mean titer; GMTR, geometric mean titer ratio; mo, month; No., number of randomized participants; NC, not computed; PVRV, purified Vero cell rabies vaccine; PVRV-NG2, next-generation purified Vero cell rabies vaccine; RVNA, rabies virus–neutralizing antibody.

^a^Per-protocol analysis set for D35.

### Safety

#### Solicited AEs

The frequencies of solicited AEs during the 7-day postvaccination period are shown in Table 2. In the PVRV-NG2 and PVRV groups, the rate of solicited injection site AEs was 37.8% (95% CI, 29.6%–46.5%) and 39.4% (95% CI, 27.6%–52.2%), respectively. Pain was the most commonly reported solicited injection site reaction in both groups (Supplementary Table 4). Most solicited injection site reactions occurred within 3 days after injection, resolved within 1–3 days, and were of grade 1 intensity. The frequency of injection site pain decreased after each dose. In the PVRV-NG2 and PVRV groups, the rate of solicited systemic AEs was 34.8% (95% CI, 26.8%–43.5%) and 21.2% (95% CI, 12.1%–33.0%), respectively (Table 2). The most common solicited systemic AE was myalgia, with rates of 30.4% (95% CI, 22.8%–38.9%) and 21.2% (95% CI, 12.1%–33.0%) in the PVRV-NG2 and PVRV groups, respectively (Supplementary Table 5).

**Table 2.** Safety Overview After Any Vaccine Injection—Safety Analysis Set

	PVRV-NG2 + HRIG (No. = 135)			PVRV + HRIG (No. = 66)		
Period/participants experiencing at least 1 event	n/M	%	(95% CI)	n/M	%	(95% CI)
Within 30 min after any vaccine injection						
Immediate unsolicited AE	0/135	0	(0–2.7)	0/66	0	(0–5.4)
Immediate unsolicited AR	0/135	0	(0–2.7)	0/66	0	(0–5.4)
Solicited reaction within 7 d after any vaccine injection	58/135	43.0	(34.5–51.8)	27/66	40.9	(29.0–53.7)
Solicited injection site reaction	51/135	37.8	(29.6–46.5)	26/66	39.4	(27.6–52.2)
Grade 3 injection site reaction	0/135	0	(0–2.7)	0/66	0	(0–5.4)
Solicited systemic reaction	47/135	34.8	(26.8–43.5)	14/66	21.2	(12.1–33.0)
Grade 3 systemic reaction	2/135	1.5	(0.2–5.2)	0/66	0	(0–5.4)
Up to 28 d after any vaccine injection						
Unsolicited AE	27/135	20.0	(13.6–27.7)	6/66	9.1	(3.4–18.7)
Unsolicited AR	1/135	0.7	(0–4.1)	1/66	1.5	(0–8.2)
Unsolicited injection site AR	1/135	0.7	(0–4.1)	1/66	1.5	(0–8.2)
Unsolicited systemic AE	27/135	20.0	(13.6–27.7)	5/66	7.6	(2.5–16.8)
Unsolicited systemic AR	0/135	0	(0–2.7)	0/66	0	(0–5.4)
SAE	0/135	0	(0–2.7)	0/66	0	(0–5.4)
Death	0/135	0	(0–2.7)	0/66	0	(0–5.4)
During the study						
SAE	1/135	0.7	(0–4.1)	0/66	0	(0–5.4)
Death	0/135	0	(0–2.7)	0/66	0	(0–5.4)
AESI	0/135	0	(0–2.7)	0/66	0	(0–5.4)

Abbreviations: AE, adverse event; AESI, adverse event of special interest; AR, adverse reaction; d, days; HRIG, human rabies immunoglobulin; M, number of participants with available data for the relevant end point; n, number of participants experiencing the relevant end point; No., number of participants in the safety analysis set; PVRV, purified Vero cell rabies vaccine; PVRV-NG2, next-generation purified Vero cell rabies vaccine; SAE, serious adverse event.

#### Unsolicited AEs

The rate of unsolicited AEs during the 28-day postvaccination period is shown in Table 2. There were no unsolicited AEs during the 30-minute postvaccination period. In the PVRV-NG2 and PVRV groups, the rate of unsolicited AEs was 20.0% (95% CI, 13.6%–27.7%) and 9.1% (95% CI, 3.4%–18.7%), respectively; only 1 participant per group experienced an unsolicited AR (considered related to the vaccine by the investigator). There were no AEs that led to study discontinuation, and no grade 3 unsolicited AEs were reported. There was 1 non-vaccine-related SAE during the study (suspected exposure to communicable disease [rabies]). There were no SAEs within 28 days of vaccination, and no AESIs or deaths were reported.

## DISCUSSION

In this descriptive study, immunogenicity outcomes were consistent between a PVRV-NG2 candidate vaccine and a licensed PVRV vaccine administered using a simulated PEP Zagreb regimen with co-administered HRIG at D0, and all participants had titers ≥0.5 IU/mL after the third dose of vaccine. In both vaccine groups, GMTs peaked after dose 3 and remained 15-fold higher at D90 compared with D0. The immunogenicity results reported here are consistent with those of a previous study that reported similar immunogenicity findings (proportion of participants with RVNA titers ≥0.5 IU/mL and GMTs) between PVRV-NG2 + HRIG and PVRV + HRIG at all time points up to D42 (14 days after the fifth dose) when administered as part of a 5-dose PEP Essen regimen (D0, D3, D7, D14, D28 [1-1-1-1-1]) with co-administered HRIG at D0 [16]. That study also demonstrated the noninferiority of PVRV-NG2 + HRIG when compared with PVRV + HRIG in terms of the proportion of participants achieving RVNA titers ≥0.5 IU/mL at D28 [16].

At D14 (7 days after the second dose of PVRV-NG2 or PVRV), the proportion of participants in our study with RVNA titers ≥0.5 IU/mL was 91.0% (95% CI, 84.1%–95.6%) and 94.6% (95% CI, 85.1%–98.9%), respectively. Our results differ slightly from those of the other studies that reported almost 100% of participants achieving RVNA titers ≥0.5 IU/mL at D14 [24, 25]. Slight differences in antibody responses between studies may be due in part to the variability in RFFIT measurements between laboratories, changes in reference standards used, and assay modifications [26]. Furthermore, the use of rabies immunoglobulins can suppress antibody production, as shown in previous studies that used the Essen (5 dose) or Zagreb regimens and that included groups without rabies immunoglobulin co-administration [16, 27, 28]. In our study, all participants in the PVRV-NG2 and PVRV groups had RVNA titers ≥0.5 IU/mL after the third dose of vaccine; this is consistent with previous studies using a simulated PEP Zagreb regimen for PVRV or other licensed vaccines, with or without the use of rabies immunoglobulin (RIG) [24, 27, 29–31].

It is important to note that at D14, most participants had RVNA titers ≥0.2 IU/mL after vaccination with PVRV-NG2 and PVRV. An RVNA titer ≥0.2 IU/mL is in line with the previous US Advisory Committee on Immunization Practices (ACIP) criteria assessing a complete neutralization of the rabies virus challenge dose at a 1:5 serum dilution using RFFIT [32]; this serum dilution corresponds to ∼0.1–0.3 IU/mL (a threshold that was used until 2022), with no reports of infections among vaccinated individuals who received adequate administration of PEP or PrEP in the United States [26, 33]. Despite no specific titer value being proven to be protective, detectable RVNAs on or before D14 is considered proof of robust immunity [34], and an RVNA titer of 0.5 IU/mL by D14 postvaccination is taken as adequate proof of PEP immunity.

GMTs in both study groups remained substantially elevated compared with D0 up to D90. The effect of PVRV on immunogenicity over time is well documented [12, 27, 35]. In an earlier study that used the Essen regimen, GMTs of those vaccinated with PVRV or PVRV-NG were similar, with a marked increase after 3 vaccinations and on completion of the 5-dose regimen in those in China aged ≥10 years [11]. Furthermore, GMTs remained high at month 7 (6 months after dose 5) in the PVRV-NG2 and HDCV groups in another study using the 5-dose Essen regimen [36] and at D90 after administration of PVRV using the Zagreb regimen [27], despite reports of low GMTs at D90 with PVRV [31]. Overall, our results highlight the similarity in immunogenicity profile between PVRV-NG2 and PVRV up to D90.

Our results suggest that the immunogenicity achieved with both PVRV-NG2 and PVRV administered as a PEP 4-dose Zagreb regimen would be sufficiently robust and timely to provide protection following potential exposure to rabies. Traditionally, the 5-dose Essen regimen has been used for PEP, yet the Zagreb regimen may be considered more favorable than this regimen because it requires fewer clinic visits, which leads to higher compliance [6]. For example, in a retrospective case study in Beijing, among 2095 adults with rabies exposure for which they received a Vero cell–adapted virus vaccine by the Zagreb or Essen regimen, the completion rates were 80.41% and 68.83%, respectively [37]. Moreover, in a meta-analysis of 13 immunogenicity studies of rabies vaccines using a PEP Zagreb or 5-dose Essen regimen, at D7 the rate of participants achieving adequate RVNA titers was significantly higher with the Zagreb than the Essen regimen [25]. The Zagreb regimen has been adopted by several countries in which rabies is highly endemic, such as Bangladesh, Cambodia, China, Indonesia, Iran, Pakistan, and Sri Lanka [2, 8, 38–40]. In Thailand, a PEP 5-dose Essen regimen for IM vaccine is recommended without RIG for category II exposure (nibbles or scratches without bleeding) and with RIG for category III exposure (transdermal bites or scratches, mucous membrane contamination with saliva from licks, licks on broken skin, direct contact with bats) [5].

The safety profile of the PVRV-NG2 candidate vaccine was consistent with that of the licensed PVRV vaccine and with the established safety profile of rabies vaccines. After the first dose of PVRV-NG2 or PVRV, the most frequent solicited injection site AE was pain (37.8% and 39.4% of participants, respectively), and the most frequent solicited systemic AE was myalgia (30.4% and 21.2% of participants, respectively). The frequency of injection site pain and myalgia decreased with subsequent doses. No SAEs, grade 3 unsolicited AEs, or AEs that led to study discontinuation were reported in the current study, and the overall safety profiles of PVRV-NG2 and PVRV were similar, which is consistent with previous observations [11, 16]. In addition, the safety profile of PVRV-NG2 was shown to be similar to that of HDCV, which is consistent with these prior studies.

The main limitations of the study were that the analyses were descriptive and that we assessed the current standard of care for PEP according to the US ACIP recommendations, rather than local guidelines in Thailand. Because all participants in our study received HRIG, it was not possible to examine the immunogenicity of the Zagreb regimen without HRIG. In addition, although the WHO currently defines an adequate response to vaccination as an RVNA titer ≥0.5 UL/mL, a major limitation of any rabies vaccine study is that this surrogate marker is indicative of vaccine response but does not establish a level of protection in humans [26]. As the current study investigated IM administration of PVRV-NG2, which can lead to reduced vaccine availability and increased costs because it requires a full vial of vaccine, further research should investigate the use of intradermal administration of PVRV-NG2 whereby several recipients can receive vaccination from the same vial within the recommended stability window following reconstitution.

In conclusion, we demonstrated that PVRV-NG2 achieves a similar level of immunogenicity and has a similar safety profile as PVRV. These results thus support the use of PVRV-NG2, a highly purified, serum- and antibiotic-free candidate, to help increase access and maintain the supply of rabies vaccines.

## Supplementary Material

ofae633_Supplementary_Data

## Supplementary Data

Supplementary materials are available at *Open Forum Infectious Diseases* online. Consisting of data provided by the authors to benefit the reader, the posted materials are not copyedited and are the sole responsibility of the authors, so questions or comments should be addressed to the corresponding author.
