# Supplementary material for: Randomized Controlled Trial of the Immunogenicity and Safety of a Serum-Free Purified Vero Rabies Vaccine (PVRV-NG2) Using a Simulated Postexposure Zagreb Regimen With Human Rabies Immunoglobulin in Adults in Thailand
Source: Open Forum Infect Dis. 2024 Oct 25;11(11):ofae633. doi: 10.1093/ofid/ofae633 (PMC11561577; doi:10.1093/ofid/ofae633)
Supplement: ofae633_Supplementary_Data [file ofae633_supplementary_data.docx]

**SUPPLEMENTARY MATERIAL**

**Randomized controlled trial of the immunogenicity and safety of a serum-free purified Vero rabies vaccine (PVRV-NG2) using a simulated post-exposure Zagreb regimen with human rabies immunoglobulin in adults in Thailand**

Danaya Chansinghakul; Terapong Tantawichien; Kriengsak Limkittikul; Winai Ratanasuwan; Yuancheng Wang; Celine Petit; Francoise Guinet-Morlot; Carina Frago; Andrea-Clemencia Pineda-Peña

**Contents**

[**Supplementary Table 1.** Protocol deviations at D14 and D35 2](#_Toc171512291)

[**Supplementary Table 2.** Baseline demographics by randomized group – Full Analysis Set 3](#_Toc171512292)

[**Supplementary Table 3.** Immunogenicity – RVNA titers (rapid fluorescent focus inhibition test – IU/mL) – Full Analysis Set 4](#_Toc171512293)

[**Supplementary Table** **4.** Summary of solicited reactions within 7 days after any vaccine injection – Safety Analysis Set 6](#_Toc171512294)

[**Supplementary Table 5.** Solicited systemic reactions within 7 days after each vaccine dose – Safety Analysis Set 7](#_Toc171512295)

# **Supplementary Table 1.** Protocol deviations at D14 and D35

|  | **PVRV-NG2 (N=135)** | **PVRV  (N=66)** | **All  (N=201)** |
| --- | --- | --- | --- |
| N excluded from FAS due to protocol deviation at D14 | 24 (17.8) | 10 (15.2) | 34 (16.9) |
| Inclusion criteria not met/met one or more exclusion criteria | 1 (0.7) | 0 (0.0) | 1 (0.5) |
| First 2 doses incomplete | 1 (0.7) | 0 (0.0) | 1 (0.5) |
| Post dose serology sample not provided | 2 (1.5) | 0 (0.0) | 2 (1.0) |
| Serology sample missing/invalid | 4 (3.0) | 0 (0.0) | 4 (2.0) |
| Seropositive at D0 | 21 (15.6) | 10 (15.2) | 31 (15.4) |
| N excluded from FAS due to protocol deviation at D35 | 22 (16.3) | 11 (16.7) | 33 (16.4) |
| Inclusion criteria not met/met one or more exclusion criteria | 1 (0.7) | 0 (0.0) | 2 (1.0) |
| 3 doses incomplete | 2 (1.5) | 0 (0.0) | 2 (1.0) |
| Post dose serology sample not provided | 2 (1.5) | 0 (0.0) | 2 (1.0) |
| Serology sample missing/invalid | 2 (1.5) | 1 (1.5) | 3 (1.5) |
| Seropositive at D0 | 21 (15.6) | 10 (15.2) | 31 (15.4) |

D, day; FAS, full analysis set; N, number of randomized participants; PVRV, purified Vero cell rabies vaccine; PVRV-NG2, next-generation purified Vero cell rabies vaccine.

# **Supplementary Table 2.** Baseline demographics by randomized group – Full Analysis Set

|  | **PVRV-NG2 (N=135)** | **PVRV  (N=66)** | **All  (N=201)** |
| --- | --- | --- | --- |
| Age, Mean (SD), years | 36.6 (12.2) | 36.3 (13.0) | 36.5 (12.4) |
| Sex, n (%) |  |  |  |
| Male | 86 (63.7) | 31 (47.0) | 117 (58.2) |
| Female | 49 (36.3) | 35 (53.0) | 84 (41.8) |
| Sex ratio, Male/Female | 1.8 | 0.9 | 1.4 |
| Racial origin, n (%) |  |  |  |
| Asian | 133 (98.5) | 66 (100.0) | 199 (99.0) |
| Mixed race | 2 (1.5) | 0 | 2 (1.0) |
| Ethnicity, n (%) |  |  |  |
| Not Hispanic or Latino | 135 (100.0) | 66 (100.0) | 201 (100.0) |
| Weight, mean (SD), kg | 62.4 (11.8) | 61.8 (10.1) | 62.2 (11.2) |
| Height, mean (SD), cm | 161 (9.2) | 162 (8.8) | 161 (9.1) |

N, number of randomized participants; PVRV, purified Vero cell rabies vaccine; PVRV-NG2, next-generation purified Vero cell rabies vaccine; SD, standard deviation.

# **Supplementary Table 3.** Immunogenicity – RVNA titers (rapid fluorescent focus inhibition test – IU/mL) – Full Analysis Set

|  | **PVRV-NG2**  **(N=135)** | **PVRV (N=66)** |
| --- | --- | --- |
| **D0 (prior to vaccination)** |  |  |
| Participants with RVNA titer, n | 135 | 66 |
| Participants with RVNA titer ≥0.5 IU/mL, n | 18 | 6 |
| Participants with RVNA titer ≥0.5 IU/mL, % (95% CI) | 13.3 (8.1, 20.3) | 9.1 (3.4, 18.7) |
| GMT, IU/mL (95% CI) | 0.148 (0.125, 0.175) | 0.151 (0.113, 0.201) |
| RVNA titer range, IU/mL | 0.100–17.8 | 0.100–27.3 |
| **D14 (7 days after vaccination dose 2)** |  |  |
| Participants with RVNA titer, n | 131 | 66 |
| Participants with RVNA titer ≥0.5 IU/mL, n | 121 | 63 |
| Participants with RVNA titer ≥0.5 IU/mL, % (95% CI) | 92.4 (86.4, 96.3) | 95.5 (87.3, 99.1) |
| GMT, IU/mL (95% CI) | 4.15 (3.00, 5.73) | 4.24 (2.76, 6.52) |
| RVNA titer range, IU/mL | 0.141–1358 | 0.100–452 |
| GMTR, D14/D0 (95% CI) | 28.1 (22.5, 35.2) | 28.1 (20.6, 38.3) |
| **D35 (14 days after vaccination dose 3)** |  |  |
| Participants with RVNA titer, n | 133 | 65 |
| Participants with RVNA titer ≥0.5 IU/mL, n | 133 | 65 |
| Participants with RVNA titer ≥0.5 IU/mL, % (95% CI) | 100 (97.3, 100) | 100 (94.5,100) |
| GMT, IU/mL (95% CI) | 11.5 (9.38, 14.1) | 12.7 (9.61, 16.9) |
| RVNA titer range, IU/mL | 0.794–1146 | 2.09–888 |
| GMTR, D35/D0 (95% CI) | 78.4 (67.8, 90.7) | 83.9 (66.3, 106) |
| **D90 (up to 3 months after vaccination dose 1)** |  |  |
| Participants with RVNA titer, n | 133 | 66 |
| Participants with RVNA titer ≥0.5 IU/mL, n | 122 | 63 |
| Participants with RVNA titer ≥0.5 IU/mL, % (95% CI) | 91.7 (85.7, 95.8) | 95.5 (87.3, 99.1) |
| GMT, IU/mL (95% CI) | 2.54 (1.96, 3.28) | 3.01 (2.12, 4.27) |
| RVNA titer range, IU/mL | 0.100–712 | 0.300–467 |
| GMTR, D90/D0 (95% CI) | 17.3 (14.7, 20.4) | 20.0 (15.6, 25.6) |

CI, confidence interval; D, day; GMT, geometric mean titer; GMTR, geometric mean titer ratio; N, number of randomized participants; PVRV, purified Vero cell rabies vaccine; PVRV-NG2, next-generation purified Vero cell rabies vaccine; RVNA, rabies virus neutralizing antibody.

# **Supplementary Table** **4.** Summary of solicited reactions within 7 days after any vaccine injection – Safety Analysis Set

|  | **PVRV-NG2+HRIG (N=135)** | | | **PVRV+HRIG (N=66)** | | |
| --- | --- | --- | --- | --- | --- | --- |
| Participants experiencing at least one event/Time point | n/M | % | (95% CI) | n/M | % | (95% CI) |
| Injection site pain | 51/135 | 37.8 | (29.6, 46.5) | 26/66 | 39.4 | (27.6, 52.2) |
| Post dose 1 | 44/135 | 32.6 | (24.8, 41.2) | 22/66 | 33.3 | (22.2, 46.0) |
| Post dose 2 | 33/133 | 24.8 | (17.7, 33.0) | 16/66 | 24.2 | (14.5, 36.4) |
| Post dose 3 | 20/133 | 15.0 | (9.4, 22.3) | 11/66 | 16.7 | (8.6, 27.9) |
| Injection site erythema | 1/135 | 0.7 | (0, 4.1) | 0/66 | 0 | (0, 5.4) |
| Post dose 1 | 1/135 | 0.7 | (0, 4.1) | 0/66 | 0 | (0, 5.4) |
| Post dose 2 | 0/133 | 0 | (0, 2.7) | 0/66 | 0 | (0, 5.4) |
| Post dose 3 | 0/133 | 0 | (0, 2.7) | 0/66 | 0 | (0, 5.4) |
| Injection site swelling | 0/135 | 0 | (0, 2.7) | 0/66 | 0 | (0, 5.4) |
| Post dose 1 | 0/135 | 0 | (0, 2.7) | 0/66 | 0 | (0, 5.4) |
| Post dose 2 | 0/133 | 0 | (0, 2.7) | 0/66 | 0 | (0, 5.4) |
| Post dose 3 | 0/133 | 0 | (0, 2.7) | 0/66 | 0 | (0, 5.4) |

CI, confidence interval; HRIG, human rabies immunoglobulin; M, number of participants with available data for the relevant endpoint; n, number of participants experiencing the relevant endpoint; N, number of participants in Safety Analysis Set; PVRV, purified Vero cell rabies vaccine; PVRV-NG2, next-generation purified Vero cell rabies vaccine.

# **Supplementary Table 5.** Solicited systemic reactions within 7 days after each vaccine dose – Safety Analysis Set

|  | **PVRV-NG2+HRIG (N=135)** | | | **PVRV+HRIG (N=66)** | | |
| --- | --- | --- | --- | --- | --- | --- |
| Participants experiencing at least one event/Time point | n/M | % | (95% CI) | n/M | % | (95% CI) |
| Fever | 1/135 | 0.7 | (0, 4.1) | 0/66 | 0 | (0, 5.4) |
| Post dose 1 | 1/135 | 0.7 | (0, 4.1) | 0/66 | 0 | (0, 5.4) |
| Post dose 2 | 1/133 | 0.7 | (0, 2.7) | 0/66 | 0 | (0, 5.4) |
| Post dose 3 | 1/133 | 0.7 | (0, 2.7) | 0/66 | 0 | (0, 5.4) |
| Headache | 21/135 | 15.6 | (9.9, 22.8) | 4/66 | 6.1 | (1.7, 14.8) |
| Post dose 1 | 11/135 | 8.1 | (4.1, 14.1) | 3/66 | 4.5 | (0.9, 12.7) |
| Post dose 2 | 13/133 | 9.8 | (5.3, 16.1) | 3/66 | 4.5 | (0.9, 12.7) |
| Post dose 3 | 4/133 | 3.0 | (0.8, 7.5) | 0/66 | 0 | (0, 5.4) |
| Malaise | 26/135 | 19.3 | (13.0, 26.9) | 6/66 | 9.1 | (3.4, 18.7) |
| Post dose 1 | 17/135 | 12.6 | (7.5, 19.4) | 4/66 | 6.1 | (1.7, 14.8) |
| Post dose 2 | 11/133 | 8.3 | (4.2, 14.3) | 3/66 | 4.5 | (0.9, 12.7) |
| Post dose 3 | 9/133 | 6.8 | (3.1, 12.5) | 2/66 | 3.0 | (0.4, 10.5) |
| Myalgia | 41/135 | 30.4 | (22.8, 38.9) | 14/66 | 21.2 | (12.1, 33.0) |
| Post dose 1 | 29/135 | 21.5 | (14.9, 29.4) | 13/66 | 19.7 | (10.9, 31.3) |
| Post dose 2 | 21/132 | 15.9 | (10.1, 23.3) | 6/66 | 9.1 | (3.4, 18.7) |
| Post dose 3 | 14/133 | 10.5 | (5.9, 17.0) | 2/66 | 3.0 | (0.4, 10.5) |

CI, confidence interval; HRIG, human rabies immunoglobulin; M, number of participants with available data for the relevant endpoint; n, number of participants experiencing the relevant endpoint; N, number of participants in Safety Analysis Set; PVRV, purified Vero cell rabies vaccine; PVRV-NG2, next-generation purified Vero cell rabies vaccine.
